# A functional cell-based bioassay for assessing adrenergic autoantibody activity in postural tachycardia syndrome

**DOI:** 10.1016/j.jtauto.2019.100006

**Published:** 2019-06-20

**Authors:** Thariq Badiudeen, Elizabeth A. Forsythe, Graham Bennett, Hongliang Li, Xichun Yu, Marci Beel, Zachary Nuss, Kenneth E. Blick, Luis E. Okamoto, Amy C. Arnold, Sachin Y. Paranjape, Bonnie K. Black, Connor Maxey, David C. Kem, Satish R. Raj

**Affiliations:** a Department of Cardiac Sciences, Libin Cardiovascular Institute of Alberta, University of Calgary, Calgary, T2N 4N1, Canada; b Department of Medicine, University of Oklahoma Health Sciences Center, Oklahoma City, OK, 73104, USA; c Department of Pathology, University of Oklahoma Health Sciences Center, Oklahoma City, OK, 73104, USA; d Autonomic Dysfunction Center, Department of Medicine, Vanderbilt University Medical Center, Nashville, TN, 37232, USA; e Neural & Behavioral Sciences, Penn State College of Medicine, Hershey, PA, 17033, USA

**Keywords:** Postural tachycardia syndrome, Orthostatic tachycardia, Heart rate, Autoantibody, Adrenergic receptor

## Abstract

**Background:**

Activating autoantibodies (AAb) to adrenergic receptors (AR) have previously been reported in patients with postural tachycardia syndrome (POTS). These AAb may contribute to a final common pathway for overlapping disease processes, reflecting a possible autoimmune contribution to POTS pathophysiology. In prior studies, measurement of AAb activity was inferred from costly, low-throughput, and laborious physiological assays. In the present study, we developed and validated an alternative cell-based bioassay for measuring AAb activity in serum by means of pre-treatment with monoamine oxidase (MAO).

**Methods:**

A total of 37 POTS patients and 61 sex-matched healthy control participants were included. Serum was pre-treated with MAO to remove endogenous catecholamines that could falsely inflate AR activation by AAb. A receptor-transfected cell-based bioassay was used to detect presence of α1AR-AAb and β1AR-AAb in serum.

**Results:**

MAO effectively degraded catecholamines as demonstrated by suppression of norepinephrine-induced α1AR activation in POTS (6.4 ​± ​0.7 vs. 5.5 ​± ​0.9; *P* ​= ​0.044) and in controls (4.1 ​± ​0.5 vs. 3.9 ​± ​0.6; *P* ​= ​0.001). Mean activity values were greater in the POTS vs. Controls for α1AR-AAb (6.2 ​± ​1.2 vs. 5.3 ​± ​1.0; *P* ​< ​0.001) and β1AR-AAb (5.7 ​± ​1.8 vs. 4.1 ​± ​0.9; *P* ​< ​0.001). Compared to controls, more POTS patients were positive for α1AR-AAb activity (22% vs 4%; P ​= ​0.007) and β1AR-AAb activity (52% vs. 2%; P ​< ​0.001).

**Conclusions:**

The co-presence of norepinephrine in serum samples can artifactually elevate α1AR and β1AR activity, which can be avoided by serum pre-treatment with MAO. Using this novel bioassay, we show that POTS patients have increased α1AR-AAb and β1AR-AAb activity compared to healthy controls in the largest POTS cohort reported to-date.

## Highlights

• Catecholamines can exist in stored frozen serum samples, even without antioxidants or preservatives.
• MAO pre-treatment removes interference by endogenous catecholamines in serum.
• POTS patients show elevated α1AR and β1AR activation by autoantibodies vs. Controls.

## 1 Introduction

Postural tachycardia syndrome (POTS) is a disorder of the cardiovascular system characterized by an excessive and sustained increase in heart rate upon standing [1], [2], [3]. It is estimated that 500,000–3,000,000 Americans suffer from POTS [3], [4]. POTS patients have a reduced quality of life [5], in addition to limited activities of daily living [6]. The pathophysiology of POTS is not well understood, and is thought to reflect the integration of multiple pathophysiological processes, including autoimmune, hyperadrenergic, and hypovolemic mechanisms [3]. We have previously identified a strong relationship between the presence of activating autoantibodies (AAb) to α1-adrenergic (α1AR) and β1-adrenergic (β1AR) receptors and POTS patients [7], [8]. These data corroborate earlier hypotheses that adrenergic AAb might exist and lead to a hyperadrenergic state in some patients with POTS that drives the tachycardia [9], [10], [11].

Recent studies examining an antiadrenergic autoimmune role in POTS, however, have utilized modest numbers of POTS patients that require replication in larger cohorts [7], [8]. Furthermore, measurement of AAb activity in these previous studies was challenging and had numerous limitations. While enzyme-linked immunosorbent assay (ELISA) techniques can identify the presence of autoantibody fragments, they are limited by their narrow range of detection [12], low reproducibility [13], [14], and most importantly they give no measurement of Ab activity or function. Previous work by our group [7], [8] made use of a rat cremaster arteriole assay to gain a functional measure of α1AR-AAb-mediated contractility. Unfortunately, the rat cremaster arteriole assay was expensive, time consuming, and dependent on technical expertise that precludes widespread use.

Therefore, in the present study, we developed a high-throughput and user-friendly assay that estimates AAb activity in serum. Previous work has shown that up to 50% of patients with POTS exhibit elevated circulating levels of plasma norepinephrine (NE) [2]. Indeed, high circulating NE has the potential to interfere with the physiological bioassays employed in the current study, given that NE is the natural ligand for both α1AR and β1AR. In the current study, we sought to assess feasibility of a high throughput functional assay for AR-AAb activity in POTS. We (HL) developed a protocol that involved pre-treatment of sera with monoamine oxidase (MAO) to degrade any endogenous catecholamines prior to the assays. We tested the hypothesis that serum α1AR and β1AR activity would be significantly lower following MAO pre-treatment, and that serum α1AR-AAb and β1AR-AAb activity would be higher in POTS patients compared with controls using this more exacting assay.

## 2 Methods

### 2.1 Study participants

A diagnosis of POTS was defined as a chronic disorder with a rise in heart rate of ≥30 beats/min within 10 ​min upon standing, as well as symptoms of cerebral hypoperfusion or sympathetic activation upon standing, such as tremor, asthenia, blurred vision, palpitations, or mental fogging,; with the absence of orthostatic hypotension (≥20 ​mmHg drop in systolic blood pressure) [1], [15]. POTS patients were enrolled from attendees of the 2014 Dysautonomia International Patient Meeting. POTS patients were required to be between 18 and 60 years old and have physician-confirmed diagnosis of POTS.

Healthy control participants were recruited at Vanderbilt University Medical Center (VUMC) in Nashville, TN, USA and at Oklahoma University Health Sciences Center (OUHSC) in Oklahoma City, OK, USA. All healthy participants were screened to exclude personal or family history of autoimmune disease or individual history of syncopal events, were free from systemic illness and were not taking any medications that could alter hemodynamics. All participants were at least 18 years of age and were not current smokers, pregnant, or endurance-trained athletes.

**Fig. 1:**
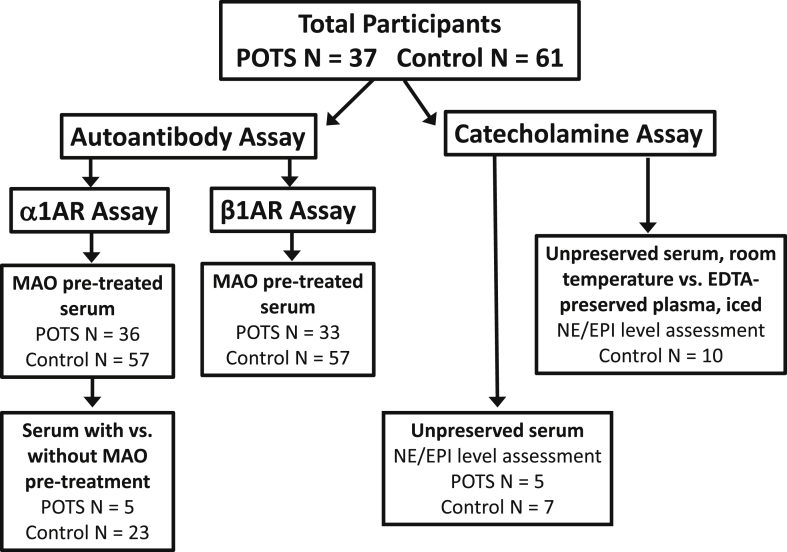
Graphical representation of all POTS patients and control participants in the entire study, and by each assay conducted.

From all participant recruitment sites (Dysautonomia International Patient Meeting, VUMC, and OUHSC), we recruited a total of 37 POTS patients and 61 Controls. Of this cohort, autoantibody assays were performed in 36 POTS patients and 57 controls. The remaining participant samples were included for the catecholamine assays only. The participant flow is outlined in Fig. 1.

This protocol was approved by the Vanderbilt University Institutional Review Board (IRB # 140671) and the Oklahoma University Institutional Review Board (IRB #4351). Each participant gave their written informed consent.

### 2.2 Clinical protocol

POTS patients were studied at the Dysautonomia International Patient Meeting (near Washington DC, USA). Whole blood was drawn while patients were seated by direct forearm venipuncture. Samples were allowed to clot at room temperature, spun, serum was harvested, and stored at −80 ​°C and shipped to VUMC on dry ice. For VUMC control participants, whole blood was drawn while seated by direct forearm venipuncture. Samples were allowed to clot at room temperature, spun, and serum was harvested using a similar protocol as for POTS patients. All POTS patient samples and VUMC control samples were stored at −80 ​°C at VUMC before being transferred to OUHSC in a blinded fashion for antibody activity assay.

For OUHSC control participants, whole blood was collected via an indwelling intravenous catheter after participants were supine for 15 ​min. Samples for catecholamines were also collected in ethylenediamine tetraacetic acid (EDTA) tubes on ice, separated and stored at −80 ​°C. Blood was allowed to clot in tubes at room temp for 2 ​h subsequently separated and also stored at −80 ​°C.

### 2.3 Laboratory protocols

All laboratory assays were performed at the OUHSC labs.

#### 2.3.1 Antibody activity assays

Serum activation of α1AR or β1AR in α1AR- or β1AR-NFAT-bla CHO–K1 cells was assessed using the GeneBLAzer FRET-based β-lactamase reporter assay (Invitrogen) according to manufacturer's instructions. Briefly, cells were plated in 384-well plates and incubated overnight. The β-lactamase substrate CCF4-AM (LiveBLAzer-FRET B/G Loading Kit, Invitrogen) was then added and incubated for 2 ​h. The plates were read using a fluorescent microplate reader (BioTek Synerg 2 Multi-Detection Microplate Reader). All samples were assayed in triplicate. Negative (buffer) controls were included in each assay. Outputs were calculated as the ratio of the emissions 460/530 ​nm (blue/green) after subtraction of the background values and expressed as fold-increase over baseline.

#### 2.3.2 Catecholamine interference and adjustment

Following an initial set of assays, concerns were raised about interference from endogenous epinephrine (EPI) and NE. To address these concerns, serum samples that had been allowed to sit at room temp for 2 ​h before storage at −80C were thawed and sent for catecholamine assay via high pressure liquid chromatography by a commercial laboratory (LabCorp, Burlington, NC). NE and EPI levels were assessed in serum collected from seven control participants and five POTS patients. Detectable EPI levels were present in the serum of five control participants and three POTS patients.

We then wanted to assess whether catecholamine levels from non-preserved serum samples were comparable to those from “properly” collected plasma samples. To address this concern, NE and EPI were measured in both serum and plasma samples from 10 control participants. Serum was stored at room temperature without EDTA for 2 ​h before processing. Plasma was iced with EDTA for 2 ​h before processing. In both cases, processing was performed by a commercial laboratory (LabCorp, Burlington, NC).

#### 2.3.3 MAO incubation prior to AAb activity assays

A total set of 5 POTS patients and 23 control participants were assayed both with and without MAO incubation. These assays were compared to discern the level of α1AR activation caused by these endogenous catecholamines and the efficacy of using MAO to remove the interference.

Following this, the AAb assays were modified such that serum samples were pre-incubated with 30 ​μg/mL monoamine oxidase A (MAO; Sigma-Aldrich, Carlsbad, CA) at 37 ​°C for 2 ​h, added to cells, and incubated for 5 ​h prior to addition of β-lactamase substrate CCF4-AM. After MAO pre-treatment, serum samples were analyzed for activation of α1AR (POTS patients n ​= ​36; control participants n ​= ​57) and β1AR (POTS patients n ​= ​33; control participants n ​= ​57). These data are reported in this paper.

POTS patients and control participants were deemed to have “elevated” α1AR-AAb or β1AR-AAb activity if their individual values were greater than 2 standard deviations above the mean value in the healthy control group.

### 2.4 Statistical analysis

Data are expressed at mean ​± ​SD. Appropriateness of assumption of normal distributions for the continuous variables was confirmed by D'Agostino-Pearson omnibus normality test. Student's t-tests were used to compare between groups and paired t-tests were used to compare within participants (before and after MAO incubation). The dichotomous threshold for defining whether a subject had elevated levels of antibody activity was defined as 2 SD above the mean values in the healthy control group. Between group differences in antibody activity meeting threshold was analyzed with a Fisher's exact test. Statistical significance was set at p ​< ​0.05. Statistical analysis was performed using SPSS version 24 (IBM Corporation, Armonk, NY). Figures were created using GraphPad Prism 6.0 (GraphPad Software, San Diego, CA).

## 3 Results

### 3.1 Study participants

Table 1 outlines the demographic profiles of POTS patients and control participants. Fig. 1 displays provides a visual representation of the total number of POTS patients and control participants, as well as a breakdown by each of the different assays performed. Among the participants with autoantibody assessments, POTS patients were younger than control participants (26 ​± ​4 years vs. 30 ​± ​11 years, P ​= ​0.018). The majority of both POTS patients and control participants were female (100% vs. 98%; P ​= ​1.0). Among patients with complete information, there was a non-significant trend towards more POTS patients reporting a family member diagnosed with POTS than controls (11.1% vs. 3.1%; P ​= ​0.07). Two POTS patients and one control participant had missing information regarding a family history of autoimmune disease. Among those with complete information, significantly more POTS patients reported a family member with an autoimmune disease than control participants (35.3% vs. 7.1%; P ​= ​0.001).

**Table 1:** Demographic profiles of patients and controls with autoantibody assays.

	POTS (n ​= ​36)	Controls (n ​= ​57)	*P* Value
Age (years±SD)	26 ​± ​4	30 ​± ​11	0.02
Females (n, %)	36 (100%)	56 (98%)	1
Family member with POTS (n, %)	4 (11%)^A^	1 (3%)^B^	0.07
Family member with autoimmune disease (n, %)	12 (35%)^C^	4 (7)^D^	0.001

### 3.2 Catecholamines in unpreserved frozen serum samples

Measurable levels of NE were found in unpreserved frozen serum samples from seven control participants (621 ​± ​165 ​pg/ml) and five POTS patients (353 ​± ​296 ​pg/ml). Additionally, measurable levels of EPI were found in unpreserved frozen serum samples from five control participants (38 ​± ​20 ​pg/ml) and three POTS patients (32 ​± ​30 ​pg/ml). The EPI level was below detection in the remaining POTS patients and control participants. This was in the absence of precooling, antioxidants and preservatives.

### 3.3 Catecholamines in serum and plasma samples

**Fig. 2:**
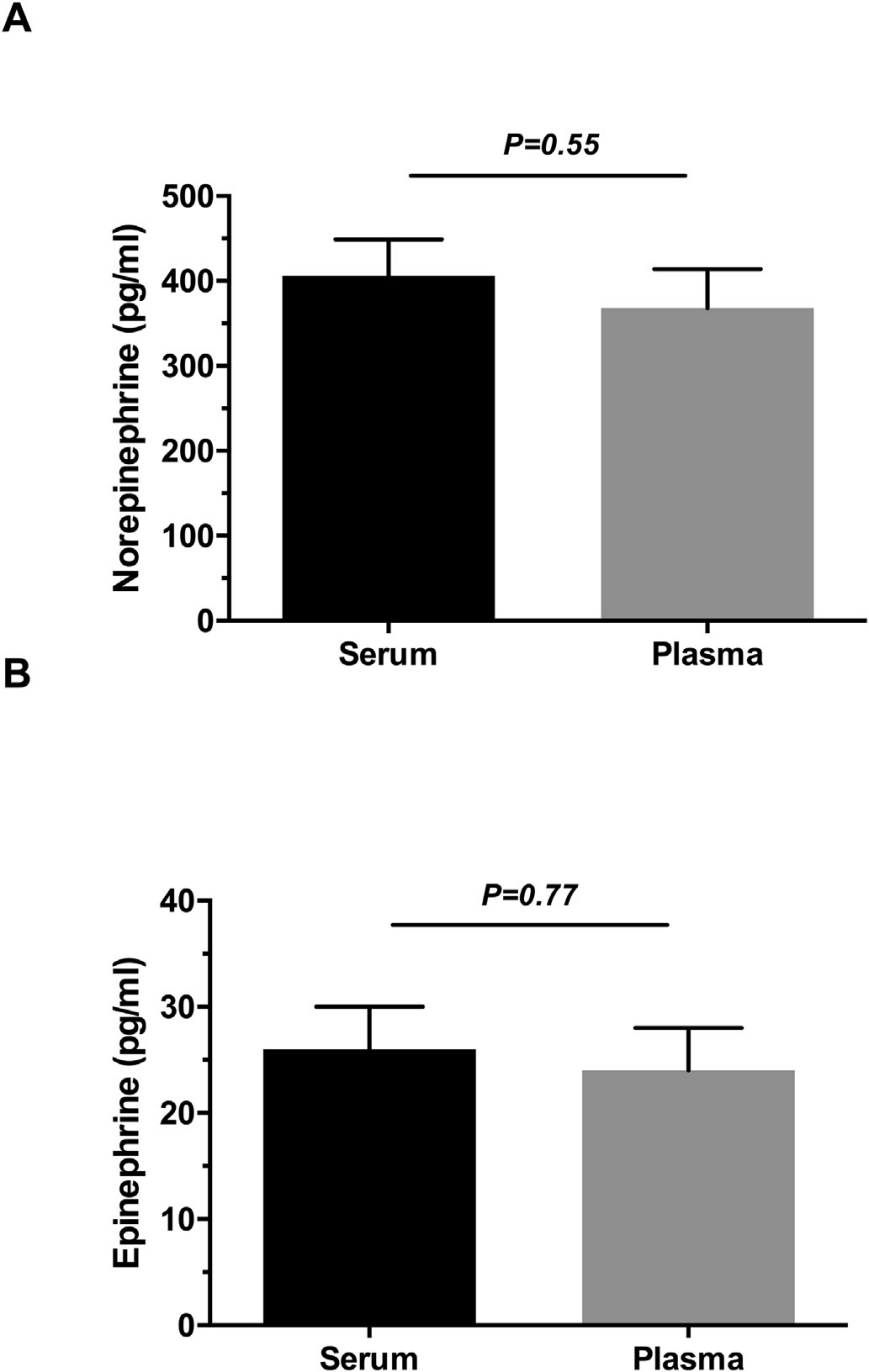
Comparison of recumbent serum and plasma norepinephrine (**Panel A**) and epinephrine (**Panel B**) in 10 control females age 20–30. Samples were drawn simultaneously. Serum was allowed to stand for 2 ​h on a counter at room temperature prior to processing. Plasma was iced and in EDTA.

Unpreserved serum and EDTA-preserved plasma NE levels assayed from 10 control participants after sitting on a bench for about 2 ​h were not statistically significant from each other (406 ​± ​137 ​pg/ml vs. 368 ​± ​145 ​pg/ml, respectively; P ​= ​0.55; Fig. 2A). Identically collected samples, showed similar levels of EPI in unpreserved serum and EDTA-preserved plasma (26 ​± ​13 ​pg/ml vs. 24 ​± ​13 ​pg/ml, respectively; P ​= ​0.77; Fig. 2B).

### 3.4 Comparison of ***α***1AR-AAb activity with and without MAO pre-treatment

**Fig. 3:**
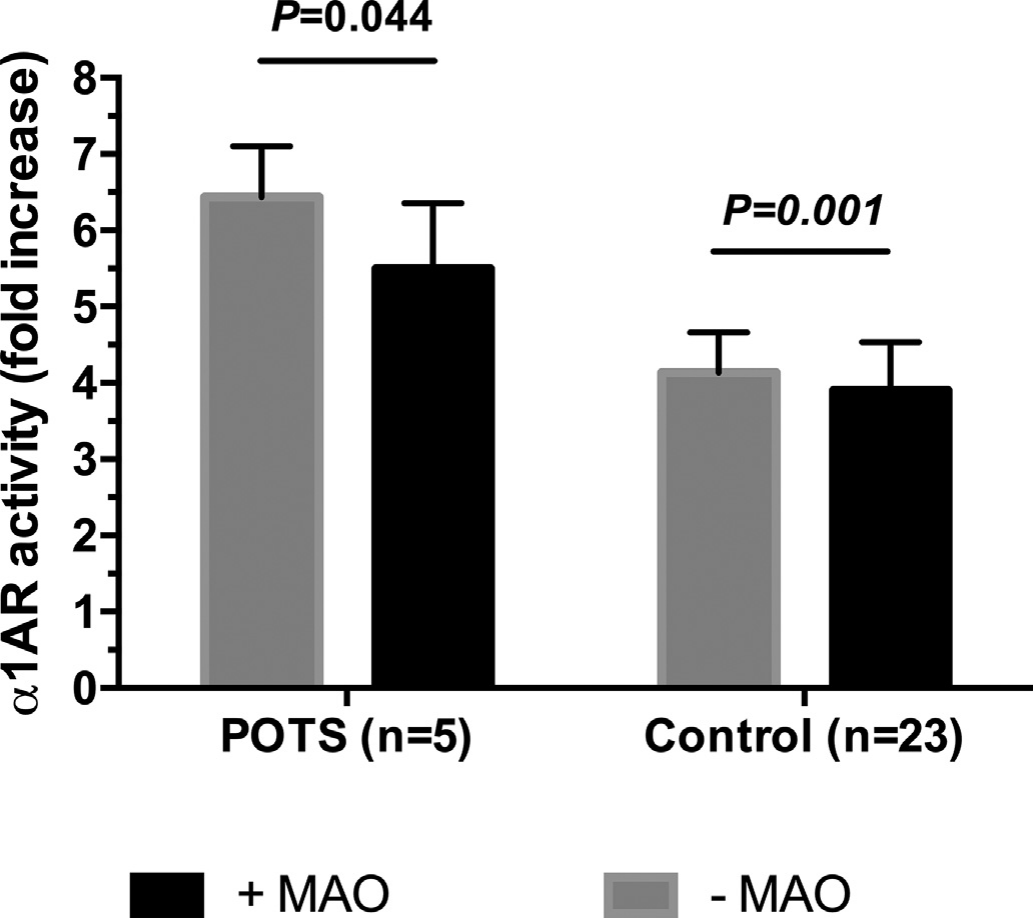
Serum autoantibody-induced α1AR activation in cell-based assays with and without MAO pre-treatment in POTS patients and healthy control participants.

Serum samples from both POTS and control groups showed significantly decreased levels of α1AR activation after pre-treatment of sera with MAO (Fig. 3). Serum sampled from POTS patients showed a decrease in α1AR-AAb activity after treatment with MAO (n ​= ​5; 6.4 ​± ​0.7 vs. 5.5 ​± ​0.9; *P* ​= ​0.044). There was a smaller, but similar, decrease in α1AR-AAb activity in the healthy control group following treatment with MAO (n ​= ​23; 4.1 ​± ​0.5 vs. 3.9 ​± ​0.6; *P* ​= ​0.001). Even when the single male control participant from this group was removed, mean values and significance level of α1AR-AAb activity before and after MAO pre-treatment were not appreciably different.

### 3.5 α1AR-AAb and β1AR-AAb activity in MAO pre-treated POTS patients and control participants

**Fig. 4:**
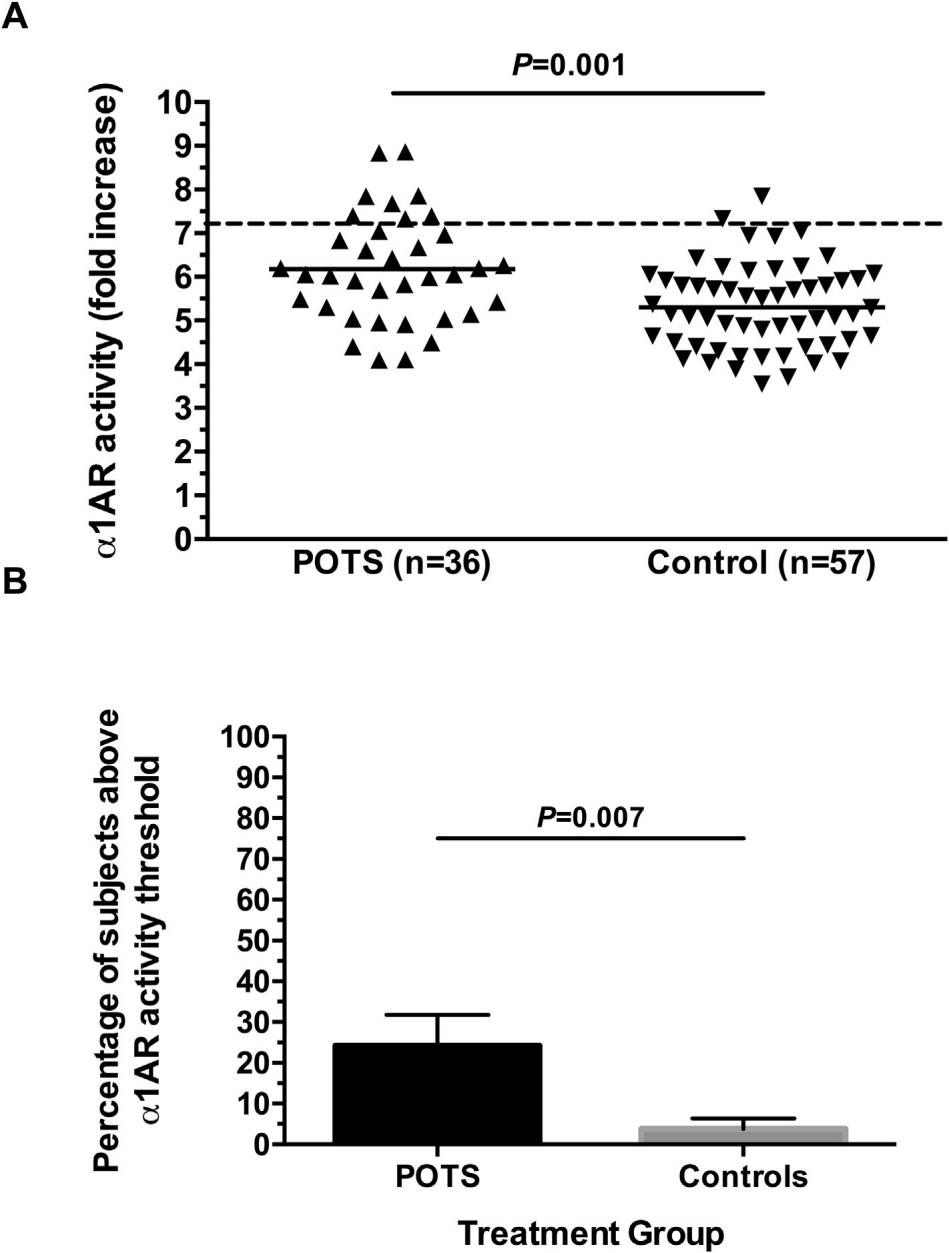
**Panel A -** Serum autoantibody-induced α1AR activation in cell-based assays. Individual participant values are presented. There was a significant increase in mean α1AR-activating autoantibody activity in the POTS group compared to Controls. Values are expressed as fold increase over buffer baseline. The dashed line represents the threshold for “elevated activity” (defined as 2SD above the mean control value). **Panel B** – The percentage of POTS patients and Control participants with “elevated” α1AR-activating autoantibody activity is shown.

**Fig. 5:**
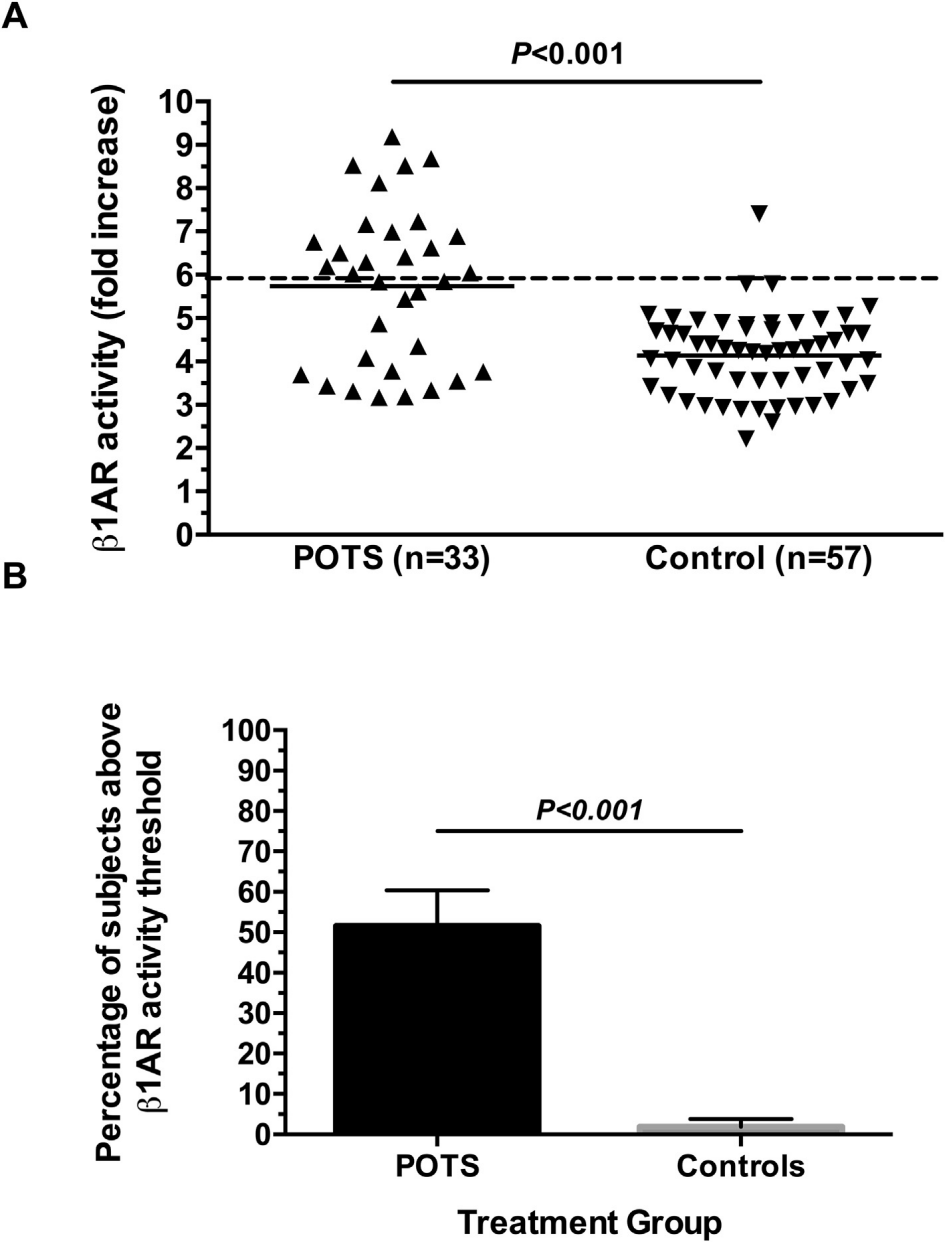
**Panel A -** Serum autoantibody-induced β1AR activation in cell-based assays. Individual participant values are presented. There was a significant increase in mean β1AR-activating autoantibody activity in the POTS group compared to Controls. Values are expressed as fold increase over buffer baseline. The dashed line represents the threshold for “elevated activity” (defined as 2SD above the mean control value). **Panel B** – The percentage of POTS patients and Control participants with “elevated” β1AR-activating autoantibody activity is shown.

The activities of α1AR-AAb and β1AR-AAb were examined using serum pretreated with MAO in cell-based bioassays in order to remove the effects of endogenous catecholamines. The α1AR-AAb activity was significantly higher in POTS patients than healthy control participants (6.2 ​± ​1.2 vs. 5.3 ​± ​1.0; *P* ​< ​0.001; Fig. 4A). Looking at individual participants, 22% of POTS patients (8/36) but only 4% of healthy controls (2/57) had elevated α1AR-AAb activity (P ​= ​0.007; Fig. 4B). The β1AR-AAb activity was significantly higher in POTS patients than healthy control participants (5.7 ​± ​1.8 vs. 4.1 ​± ​0.9; *P* ​< ​0.001; Fig. 5A). Looking at individual participants, 52% of POTS patients (17/33) but only 2% of healthy controls (1/57) had elevated β1AR-AAb activity (*P* ​< ​0.001; Fig. 5B). Even when the single male control participant from this group was removed, α1AR-AAb and β1AR-AAb activities were not appreciably different. This was also true for the number of POTS patients and control participants above threshold for α1AR-AAb and β1AR-AAb activity.

## 4 Discussion

These data represent the largest POTS patient cohort to have functional adrenergic AAb activity assessed to date. We found that as a group, POTS patients had greater agonist activity at both α1AR and β1AR. These responses reinforce previous studies identifying the presence of AAb directed towards cardiovascular G protein-coupled receptors (GPCR) in some participants with POTS.

### 4.1 Endogenous catecholamines and adrenergic receptor AAb activity assay

One challenge with measuring functional AAb activity is that it can be falsely elevated by endogenous ligands to that same receptor in cell-based assays. Catecholamines are considered to be generally unstable compounds, and can be rapidly oxidized under neutral conditions without use of an antioxidant, like glutathione or ascorbic acid [16]. One would then expect that catecholamines would degrade in serum that was frozen without preservatives. Surprisingly, we found that unpreserved deep-frozen serum samples still contained significant amounts of NE and EPI; this was also true of serum samples left to sit on a counter at room temperature, or just over ice, for a few hours (Fig. 2). Our group has previously examined two relatively small cohorts of POTS patients; we showed that a significant number of patients presented with adrenergic AAb activity [7], [8]. This finding has led to increased interest in the potential causative role that AAb play in this disease whose etiology has yet to be fully uncovered. The finding for endogenous catecholamines in our unpreserved frozen serum samples raises the possibility that prior findings might have included an artifactual component from the endogenous catecholamines.

To address this potential source of error, we developed a simple, high-throughput technique to remove interference of endogenous catecholamines by pre-treating serum with MAO. We employed this novel technique on a larger cohort of POTS patients than has been reported in all prior AAb reports of POTS patients combined.

MAO plays an important role in the degradation of NE *in vivo*. Our technique of pre-treating serum samples with 30 ​μg/mL MAO for 2 ​h prior to assay, was shown to significantly reduce measured α1AR activity. Fig. 3 shows reduced levels of α1AR activity in both POTS patients and healthy control participants after MAO pre-treatment. We attribute this reduced α1AR activity to efficacy of MAO in removing endogenous catecholamines. This technique is particularly well-suited in the context of POTS patients, some of whom have been found to have elevated circulating endogenous NE levels in excess of 600 ​pg/mL, especially on standing [1], [17]. Our findings suggest that isolating the contribution of AAb towards a hyperadrenergic state in POTS requires removal of interference by endogenous catecholamines from serum. We believe the use of MAO provides an efficient and cost-effective alternative in measuring functional activity of serum adrenergic AAb without the need for more time-intensive and expensive techniques such as IgG purification.

### 4.2 Adrenergic receptor AAb activity in POTS

The present findings for increased α1AR-AAb and β1AR-AAb activity in POTS patients relative to controls are concordant with prior studies [7], [8]. The current study has studied the largest cohort of POTS patients to date. Importantly, the observed increased α1AR and β1AR activity occurred even after MAO pre-treatment, so these were not artifacts of endogenous catecholamines. These consistent findings, with an even more rigorous method, add further support to the hypothesis that some POTS patients have excessive adrenergic receptor AAb activity.

There remain many unanswered questions regarding adrenergic receptor AAb and POTS. The first question is whether the mere presence of AAb, measured with an ELISA assay, is a reasonable surrogate for AAb activity as measured by a functional assay (as in this study). ELISA assays are cheaper and simpler to perform, but by their nature do not provide evidence for activity and effect. A second question is the prevalence of adrenergic receptor AAb in POTS. It is clear that they are not present in all POTS patients, but the magnitude of importance of this mechanism is not yet clear. A third, and perhaps most important, question is whether the *in vitro* adrenergic receptor AAb activity assays reflect altered adrenergic receptor activity in humans.

The altered AR-AAb activity is a complex issue. The direct (orthosteric) effect of the adrenergic AAb activity on its target receptor is reported as **the** outcome for each of our studies.

We previously have demonstrated differing allosteric effects of α1AR-AAb and β1AR-AAb from POTS patients [7], [8]. The α1AR-AAb produce partial antagonism to the α1AR natural ligand NE. This inadequate response to upright posture would lead to baroreceptor-enhanced sympathetic activity. In contrast, the beta1 antibodies facilitate the action of the β1AR orthosteric ligand, which would lead to enhanced chronotropic response in the heart. However, prior studies have demonstrated that this effect may not be as important as the allosteric indirect effect of the AAb on the GPCR modulation of its natural ligand [7], [8], [18]. We have demonstrated these allosteric effects are variably present for each of the AAb so far discovered [7], [8], [18]. This *allosteric impact* is potentially greater than the *direct orthosteric effects,* and we believe this allosteric modulation is involved in the pathophysiology of POTS [7]. These indirect effects are not measured with our standard assay. It is likely that the overall AAb involvement in the causation of POTS is significantly underestimated using these current orthosteric measurements. Removal of these AAb effects by pharmacological or immunological interventions will determine their ultimate role in the modifiable pathophysiology of this multifaceted disease.

### 4.3 Limitations

An important limitation of the current study concerns the possible contribution of other autoantibodies in POTS. Previous studies have shown the presence of angiotensin-receptor antibodies [18], β2AR and muscarinic cholinergic receptor antibodies [9] in patients, potentially contributing to POTS pathophysiology. These findings further reinforce the hypothesis that the syndromic presentation of POTS represents the integration of multiple pathophysiological processes may be an understatement. Additionally, POTS patients and control participants were not strictly age matched in the current study, leading to a small, but statistically significant, age difference between the groups. Differences in AR AAb activity between POTS patients and control participants may be influenced by a decline in Ab production with increasing age [19], although the age difference is so small that this seems unlikely.

## 5 Conclusions

Endogenous catecholamines can survive in a non-degraded state even in unpreserved frozen serum samples, and these catecholamines can artificially elevate adrenergic AAb activity using a cell-based assay. Pre-treatment with MAO restores normal activity. Even using this more rigorous adrenergic receptor AAb assay, POTS patients have greater adrenergic AAb activity than healthy control participants.

## Funding

This work was supported by research grants from Dysautonomia International (East Moriches, NY, USA) to SRR and DCK; 10.13039/100000002National Institutes of Health [grant numbers UL1TR000445 and R01HL128393]; and Canadian Institutes of Health Research, Ottawa, Canada [grant number MOP142426]. SRR is a Cardiac Arrhythmia Network of Canada funded investigator.

## Disclosures

SRR has been a consultant for, and received compensation from, Lundbeck LLC and GE Healthcare. SRR serves on the Medical Advisory Board of Dysautonomia International without financial compensation. None of the other authors report any disclosures.
